# Machine-learning model predicting quality of life using multifaceted lifestyles in middle-aged South Korean adults: a cross-sectional study

**DOI:** 10.1186/s12889-023-17457-y

**Published:** 2024-01-11

**Authors:** Junho Kim, Kyoungsik Jeong, Siwoo Lee, Younghwa Baek

**Affiliations:** KM Data Division, Korea Institute of Oriental Medicine, Daejeon, Republic of Korea

**Keywords:** Quality of life, Lifestyles, Prediction, Machine learning, Middle-aged

## Abstract

**Background:**

In the context of population aging, advances in healthcare technology, and growing interest in healthy aging and higher quality of life (QOL), have gained central focus in public health, particularly among middle-aged adults.

**Methods:**

This study presented an optimal prediction model for QOL among middle-aged South Korean adults (N = 4,048; aged 30–55 years) using a machine-learning technique. Community-based South Korean population data were sampled through multistage stratified cluster sampling. Twenty-one variables related to individual factors and various lifestyle patterns were surveyed. QOL was assessed using the Short Form Health Survey (SF-12) and categorized into total QOL, physical component score (PCS), and mental component score (MCS). Seven machine-learning algorithms were used to predict QOL: decision tree, Gaussian Naïve Bayes, k-nearest neighbor, logistic regression, extreme gradient boosting, random forest, and support vector machine. Data imbalance was resolved with the synthetic minority oversampling technique (SMOTE). Random forest was used to compare feature importance and visualize the importance of each variable.

**Results:**

For predicting QOL deterioration, the random forest method showed the highest performance. The random forest algorithm using SMOTE showed the highest area under the receiver operating characteristic (AUC) for total QOL (0.822), PCS (0.770), and MCS (0.786). Applying the data, SMOTE enhanced model performance by up to 0.111 AUC. Although feature importance differed across the three QOL indices, stress and sleep quality were identified as the most potent predictors of QOL. Random forest generated the most accurate prediction of QOL among middle-aged adults; the model showed that stress and sleep quality management were essential for improving QOL.

**Conclusion:**

The results highlighted the need to develop a health management program for middle-aged adults that enables multidisciplinary management of QOL.

## Introduction

Quality of life (QOL) is a broad and complicated construct encompassing important domains of daily functioning and subjective experiences; these include physical and social role functioning, physical sensation, and subjective well-being [1]. The importance of QOL—pursuing good health and not living with major illnesses—is highlighted by the 6.6-year increase in global average life expectancy, and the 1.3% decrease in premature, preventable mortality from non-communicable diseases [2, 3]. Moreover, aging well comprises minimizing physical and mental exacerbation and QOL, and the ability to enjoy a meaningful life [4].

QOL is influenced by various factors, including demographic and socioeconomic factors and comorbidities [5, 6]. Many clinical and epidemiological studies have presented evidence of the positive impact of healthy lifestyle practices on QOL. A path analysis model showed that multiple health practices—comfortable sleep, adequate physical activity, and fruit and vegetable intake—are associated with overall QOL. Adherence to a systematic lifestyle modification program leads to improvements in QOL at the one-year follow-up mark [7, 8]. A randomized controlled trial meta-analysis of lifestyle interventions in patients with metabolic syndrome showed that lifestyle interventions produce marked improvements in physical and mental QOL, compared with regular care [9]. In addition, healthy lifestyle practices and positive QOL reduce chronic disease burden and mortality risk among middle-aged adults [10, 11]. However, middle-aged adults’ healthy lifestyle practice was much lower than younger and older adults, which consequently impaired their QOL. This finding highlights the need to design age group-specific health behaviors that effectively improve QOL [7].

In recent years, machine learning (ML) has been widely used to manage big clinical data. This trend is important in understanding multiple factors’ complexity and nonlinear relations [12]. A multidimensional analysis of QOL calls for sophisticated techniques that enable automated analyses. One study identified five significant predictors of health-related QOL among older adults with chronic diseases, and confirmed that stepwise logistic regression produces an effective QOL prediction model [13]. A study on depression, which is strongly associated with QOL, attempted to establish an ML model to predict QOL using demographic and psychometric data [14]. It found that ML algorithms show superior predictive performance to conventional logistic regression and shed light on the potential of ML in individualized mental health management [14]. In Korea, a study on QOL prediction among older adults using the 36-item Short Form Survey (SF-36) conducted an elastic net-based analysis and reported that grip strength is strongly associated with older adults’ QOL [15]. As such, studies have demonstrated the effectiveness of ML in utilizing clinical data, and more active research on this topic is anticipated. We established an optimal ML model for predicting QOL, considering individual factors and multifaceted lifestyle factors of middle-aged adults in Korea. We also examined the importance and effects of various factors influencing overall, physical, and mental QOL.

## Materials and methods

### Study population and sampling

This cross-sectional study used data from the 2017–2019 Korean Medicine Daejeon Citizen Cohort study [16]. The inclusion criteria were residents of Daejeon—a city in South Korea—aged 30–55 years. The exclusion criteria were diagnosis of cancer (malignant tumor) or cardiovascular disease (e.g., myocardial infarction, angina, stroke/apoplexy) and difficulty responding to the questionnaire.

We performed multistage stratified cluster sampling. In the first stage, we divided Daejeon into five administrative units (*gu*), based on the resident registration population (approximately 610,000) aged 30–55, defined as middle-aged in this study [16, 17]. We then identified the sample size and survey point for each unit using probability proportional to size. In the second stage, we allocated samples proportional to sex (men, women) and age (30–39 years, 40–49 years, and 50–55 years) by unit. If the survey could not be performed at the identified survey point, the survey site was moved to another location within the same stratum. All participants were selected randomly at the survey point. We used a structured questionnaire in Korean that included questions on demographic factors (4 items), physical measurement and cold–heat pattern factors (4 items), lifestyle factors (13 items), and QOL, administered via one-on-one interviews.

A total of 4,063 participants completed the survey. After excluding 15 with missing data for significant variables, we analyzed data from 4,048 participants, consisting of 1,751 men and 2,297 women (ratio = 1:1.31).

### Measurements

#### Demographic factors

Individual demographic factors were sex, age, marital status, household income, and disease history. Marital status was divided into married and single (including never married, divorced, or widowed). Monthly household income was divided into ≤ 2.99, 3.00–4.99, and ≥ 5 million KRW. Chronic conditions were determined based on disease history (physician’s diagnosis of hypertension, diabetes mellitus, and hyperlipidemia) and obesity (high BMI). Based on the presence of these four conditions, chronic condition was categorized as 0 or ≥ 1 [18].

#### Physical measurement and cold–heat pattern

Individual physical measurement and cold–heat pattern factors were height, weight, BMI and cold–heat pattern identification. BMI was calculated by dividing self-reported weight (kg) by height squared (m^2^); with reference to 25 kg/m^2^, participants were categorized as being of normal weight or obese [19]. Cold–heat pattern identification is a Korean medicine pattern identified for each person, based on their preference or sensitivity to cold or hot temperatures and the temperature of their hands and feet. In traditional East Asian medicine, this pattern is used to provide health management for patients [20]. We used the cold–heat pattern identification questionnaire to analyze the cold and heat pattern scores, which consists of eight items for cold pattern and seven items for heat pattern [21]. The cold and heat pattern scores were calculated sum of the items included in each pattern on a five-point response scale from 1 to 5, with higher scores indicating closer to being in a cold or heat pattern. The questionnaire was acceptably reliable (Cronbach’s α coefficient = 0.75) and valid (72.9– 82.8% agreement, compared to two professional’s examination) [21].

#### Lifestyles

Considering alcohol consumption, we calculated the average volume of alcohol per day (g/day) based on drinking frequency (times/day), the volume of alcohol per seating (drinks/seating), and alcohol content (g/drink) for different types of alcohol in the past year. Concerning the sex-specific criteria for the average volume of alcohol per day, we divided participants into non-drinker, responsible drinker, hazardous drinker, and harmful drinker [22]. In addition, smoking status was assessed using the questions “Have you smoked more than 100 cigarettes in your lifetime?” and “Do you currently smoke?” Based on the responses, participants were categorized into current, past, and non-smoker.

Night snacking was assessed with the question, “Do you frequently eat snacks after dinner or before bed?” The responses were divided into 0–1, 2–3, and ≥ 4 times a week. The eating index was assessed using the semi-quantitative food frequency questionnaire consisting of the frequency and average intake of 34 food groups. Eating index was composed of 14 components including adequacy, moderation and balance, and the total score ranged from 0 to 100 by adding up the scores of each component using the eating index equation following the previously reported calculation method of the Korean Healthy Eating [23]. A higher eating index represents healthier eating.

Sleep in the past month was assessed using the 19-item Pittsburgh Sleep Quality Index Korean version (PSQI-K), which had high reliability and validity (Cronbach’s α coefficient = 0.84 in PSQI-K) [24]. The PSQI score ranges from 0 to 21, with the seven component scores weighted from 0 to 3 and then summed, with higher scores indicating worse sleep quality. We used sleep duration (hours) and sleep quality (PSQI score); sleep quality was divided into two groups based on a cutoff of 5 (good sleeper vs. poor sleeper) [25].

Physical activity was assessed using the Korean Global Physical Activity Questionnaire (GPAQ) developed by the World Health Organization (WHO) [26]. According to the GPAQ analysis guidebook, we calculated each domain-related physical activity (minutes/week) for work (high, moderate intensity), transport (walking or riding a bike), and recreation (high, moderate intensity), as well as sedentary time (minutes/day). We used the metabolic equivalent task (MET), which represents the intensity of physical activity, to calculate physical activity in the unit of MET-minutes/week. Finally, we assessed stress levels using the 18-item Psychosocial Well-being Index – Short Form with high internal consistency (Cronbach’s α coefficient = 0.90) [27]. Each item was evaluated on a four-point response scale from 0 to 3, with the total score ranging from 0 to 54, and a higher score indicates increased stress.

#### Health-related QOL

We assessed QOL using the Short Form Health Survey (SF-12), widely used to measure physical and mental health [28]. Although it is a short form of a 36-item survey, it is useful for clinical research and in measuring the overall impact of disease on a patient’s life [29]. This instrument consists of a physical component score (PCS) and a mental component score (MCS). The PCS includes physical functioning, role-physical, bodily pain, and general health. The MCS includes mental health, role-emotional, social functioning, and vitality. We applied norm-based scoring to the calculated PCS and MCS to convert them to a score with an average of 50 and a standard deviation of 10. The total score for each index ranges from 0 to 100, and higher scores indicate better QOL [30].

This study used three QOL indices (PCS, MCS, and total QOL [sum of PCS and MCS]). We defined a poor QOL group to establish a prediction model. Each of the three scores was divided into terciles, and a score below the lowest tercile was classified as low QOL, with any scores at or above the lowest tercile classified as high QOL. The cutoff for the lowest tercile was 49.69 for PCS, 48.46 for MCS, and 98.40 for total QOL.

### Data analysis

Data are expressed as mean and standard deviation, and frequency and percentage. We compared the general characteristics of participants between the normal and the low QOL groups using the Fisher’s exact or chi-squared test for categorical variables and independent *t*-tests for continuous variables.

We used supervised ML as a low-level ML model for QOL. The algorithms used to develop the models were decision tree, Gaussian Naïve Bayes, k-nearest neighbor, logistic regression analysis, XGBoost, random forest, and support vector machine. Min-max normalization was applied to each variable. The dataset was split into training and validation sets using six-fold cross-validation to compare model performance. The ratio of training and validation datasets was 5:1. Of the 4,048 datasets, 3,373 and 675 were used for training and validation, respectively. In addition, the 2:1 ratio of the high and low QOL groups was configured to remain the same for the training and validation datasets. To address the data imbalance, owing to the use of 1,336 datasets for the poor QOL group and 2,712 datasets for the high QOL group, we applied oversampling, performed using the synthetic minority oversampling technique (SMOTE). SMOTE generates random synthetic data based on Euclidean distance for the minority group [31]. The synthetic data exhibit features similar to existing data, which were applied in this study to compare performance before and after application. The random forest model, which showed high performance, was used to analyze the importance of variables in each of the three QOL prediction models (total QOL, PCS, and MCS).

We assessed the performance of QOL prediction models based on five indices: F1-score, accuracy, sensitivity, specificity, and area under the receiver operating characteristic (AUC). Data were analyzed using Python 3.8.10 (Python Software Foundation, PSF). The Scikit-learn library in Python was used. For analysis and comparison, we built a model using default parameters. Finally, the explanatory power of the prediction model was analyzed, using Shapley additive explanations (SHAP) [32]. SHAP analysis was performed through the SHAP library in Python, and a tree-based model was used.

## Results

### General characteristics

Table 1 shows participants’ general characteristics and the differences between the high and low total QOL groups. The two groups significantly differed in all variables, except marital status and alcohol consumption.

**Table 1:** Participants’ general characteristics

	Total	High QOL group^§^	Low QOL group^§^	*p* ^†^	Data type
Sex	4048 (100)	2712 (67.0)	1336 (33.0)		Categorical
Male	1751 (43.3)	1344 (76.8)	407 (23.2)	< 0.001	“1”
Female	2297 (56.7)	1368 (59.6)	929 (40.4)		“2”
Age (years)	43.55 ± 7.23	43.13 ± 7.31	44.42 ± 6.99	< 0.001	Continual
Body mass index	25.64 ± 27.88	24.89 ± 22.57	27.16 ± 36.30	0.015	Continual
Marital status					Categorical
Single	693 (17.1)	471 (68.0)	222 (32.0)	0.551	“0”
Married	3355 (82.9)	2241 (66.8)	1114 (33.2)		“1”
Household income (million KRW)					Categorical
< 299	718 (17.7)	349 (48.6)	369 (51.4)	< 0.001	“0”
300–499	1544 (38.1)	1026 (66.5)	518 (33.5)		“1”
> 500	1786 (44.1)	1337 (74.9)	449 (25.1)		“2”
Chronic condition					Categorical
Without	2617 (64.6)	1813 (69.3)	804 (30.7)	< 0.001	“0”
With one more	1431 (35.4)	899 (62.8)	532 (37.2)		“1”
Cold pattern score	52.35 ± 15.51	50.53 ± 15.22	56.04 ± 15.43	< 0.001	Continual
Heat pattern score	52.02 ± 16.60	51.57 ± 15.88	52.94 ± 17.94	0.014	Continual
Alcohol consumption					Categorical
Non-drinker	1477 (36.5)	927 (62.8)	550 (37.2)	0.073	“0”
Responsible drinker	2127 (52.5)	1501 (70.6)	626 (29.4)		“1”
Hazardous drinker	258 (6.4)	168 (65.1)	90 (34.9)		“2”
Harmful drinker	186 (4.6)	116 (62.4)	70 (37.6)		“3”
Smoking					Categorical
Non-smoker	2957 (73.0)	1935 (65.4)	1022 (34.6)	< 0.001	“1”
Former smoker	334 (8.3)	227 (68.0)	107 (32.0)		“2”
Current smoker	757 (18.7)	550 (72.7)	207 (27.3)		“3”
Late night eating (freq/wk)					Categorical
4–7	450 (11.1)	226 (50.2)	224 (49.8)	< 0.001	“1”
2–3	2205 (54.5)	1473 (66.8)	732 (33.2)		“2”
0–1	1393 (34.4)	1013 (72.7)	380 (27.3)		“3”
Eating index (score 0–100)	51.35 ± 10.55	51.69 ± 10.37	50.64 ± 10.86	0.003	Continual
Sleep duration (hr)	6.78 ± 1.05	6.87 ± 0.93	6.58 ± 1.22	< 0.001	Continual
Sleep quality (score 0–21)	4.46 ± 2.80	3.58 ± 2.17	6.25 ± 3.06	< 0.001	Continual
Sleep quality group					Categorical
Good sleeper	2892 (71.4)	2274 (78.6)	618 (21.4)	< 0.001	“1”
Poor sleeper	1156 (28.6)	438 (37.9)	718 (62.1)		“2”
PA score (MET-min/wk)	2581.86 ± 4036.99	2330.38 ± 3614.76	3092.36 ± 4739.89	< 0.001	Continual
Sedentary time (min/d)	444.21 ± 175.66	450.98 ± 168.64	430.46 ± 188.35	< 0.001	Continual
Work-related PA (min/wk)	268.36 ± 613.11	228.18 ± 537.96	349.92 ± 735.92	< 0.001	Continual
Transport-related PA (min/wk)	129.40 ± 245.58	117.99 ± 231.51	152.57 ± 270.44	< 0.001	Continual
Recreation-related PA (min/wk)	133.29 ± 223.66	140.17 ± 227.98	119.35 ± 213.95	0.005	Continual
Stress (score 0–54)	17.02 ± 7.11	14.48 ± 5.73	22.18 ± 6.85	< 0.001	Continual

Values are presented as *n* (%) or mean ± standard deviation. QOL, quality of life; PA, physical activity; MET: metabolic equivalent task

^†^*P*-values for continuous variables are based on independent *t*-tests; all other *P*-values for categorical variables are based on Fisher’s exact test or chi-squared test between the high and low QOL groups

^§^ High (≥ 98.4) and low (< 98.4) QOL groups, divided based on total QOL score

### Comparison of machine-learning models for predicting QOL

As shown in Table 2, we created total QOL, PCS, and MCS prediction models and compared their performances. For total QOL, the AUC of the models ranged from 0.688 to 0.747, and the XGBoost model had the highest performance. Regarding the F1-score, random forest and logistic regression models showed high performance. For PCS, the AUC of the models ranged from 0.610 to 0.658, and the random forest model also had high performance. Regarding F1-score and accuracy, the model had logistic regression analyses of 0.721 and 0.739, respectively. For MCS, the AUC ranged from 0.615 to 0.698, and XGBoost showed high performance. Regarding F1-score and accuracy, the random forest model showed high performance. Although the results varied for the target QOL, the tree-based random forest model showed good performance, overall.

**Table 2:** Comparison of ML model performance for predicting total QOL, PCS, and MCS

	F1-score		Accuracy		Sensitivity		Specificity		AUC	
	Original	SMOTE	Original	SMOTE	Original	SMOTE	Original	SMOTE	Original	SMOTE
***TOTAL***										
DecisionTree	0.724	0.745	0.725	0.744	0.579	0.711	0.796	0.769	0.688	0.740
GaussianNB	0.759	0.730	0.765	0.735	0.566	0.602	0.863	0.835	0.715	0.718
KNN	0.737	0.746	0.751	0.746	0.472	0.700	0.889	0.780	0.680	0.740
Logistic Regression	0.792	0.780	0.800	0.782	0.582	0.685	0.907	0.855	0.744	0.770
XGBoost	0.787	0.828	0.791	0.829	0.619	0.774	0.875	0.871	0.747	0.822
Random Forest	0.792	0.829	0.799	0.830	0.586	0.768	0.904	0.876	0.745	0.822
SVM	0.788	0.780	0.796	0.783	0.571	0.684	0.907	0.856	0.739	0.770
***PCS***										
DecisionTree	0.653	0.681	0.652	0.681	0.487	0.642	0.733	0.710	0.610	0.676
GaussianNB	0.706	0.676	0.716	0.685	0.465	0.514	0.838	0.812	0.652	0.663
KNN	0.689	0.715	0.706	0.714	0.395	0.687	0.858	0.734	0.626	0.711
Logistic Regression	0.721	0.711	0.739	0.716	0.423	0.579	0.893	0.819	0.658	0.699
XGBoost	0.703	0.756	0.710	0.757	0.480	0.681	0.823	0.814	0.652	0.747
Random Forest	0.719	0.778	0.735	0.780	0.435	0.701	0.882	0.838	0.658	0.770
SVM	0.719	0.699	0.736	0.707	0.429	0.539	0.886	0.833	0.657	0.686
***MCS***										
DecisionTree	0.674	0.717	0.674	0.717	0.507	0.674	0.756	0.750	0.631	0.712
GaussianNB	0.716	0.683	0.722	0.687	0.505	0.556	0.829	0.786	0.667	0.671
KNN	0.679	0.706	0.698	0.706	0.370	0.681	0.860	0.724	0.615	0.702
Logistic Regression	0.747	0.725	0.761	0.730	0.482	0.596	0.898	0.829	0.690	0.713
XGBoost	0.745	0.790	0.752	0.791	0.539	0.713	0.857	0.850	0.698	0.781
Random Forest	0.750	0.794	0.762	0.796	0.497	0.712	0.892	0.859	0.695	0.786
SVM	0.746	0.727	0.761	0.731	0.470	0.596	0.905	0.833	0.687	0.714

The results before (Original) and after (SMOTE) SMOTE application were included. SMOTE, synthetic minority oversampling technique; AUC, area under the receiver operating characteristic curve; GaussianNB, Gaussian Naïve Bayes classifier; KNN, K-nearest neighbor; XGBoost, extreme gradient boosting; SVM, support vector machine; PCS, Physical component score; MCS, Mental component score.

### Performance with and without the synthetic minority oversampling technique

**Fig. 1:**
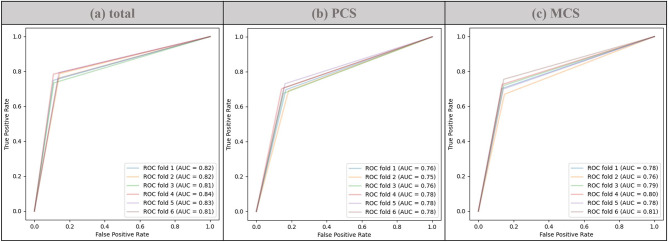
ROC curve for QOL prediction. ROC curves by fold for the random forest model for predicting total QOL (**a**), PCS (**b**), and MCS (**c**). PCS, Physical component score; MCS, Mental component score; ROC curve, Receiver Operating Characteristic curve

Table 2 shows performance comparison after using SMOTE. When total QOL was predicted after applying SMOTE, the random forest and XGBoost models had an AUC of 0.822, with the former having a higher F1-score (0.829). When PCS was predicted after applying SMOTE, the random forest model showed good performance, with an AUC of 0.770 and an F1-score of 0.778. Finally, when MCS was predicted after applying SMOTE, the random forest model showed good performance in terms of AUC (0.786), F1-score (0.794), and accuracy (0.796). Figure 1 shows the ROC curve, by fold-in cross-validation, for predicting the three types of QOL after applying SMOTE.

### Key factors in predicting QOL

**Fig. 2:**
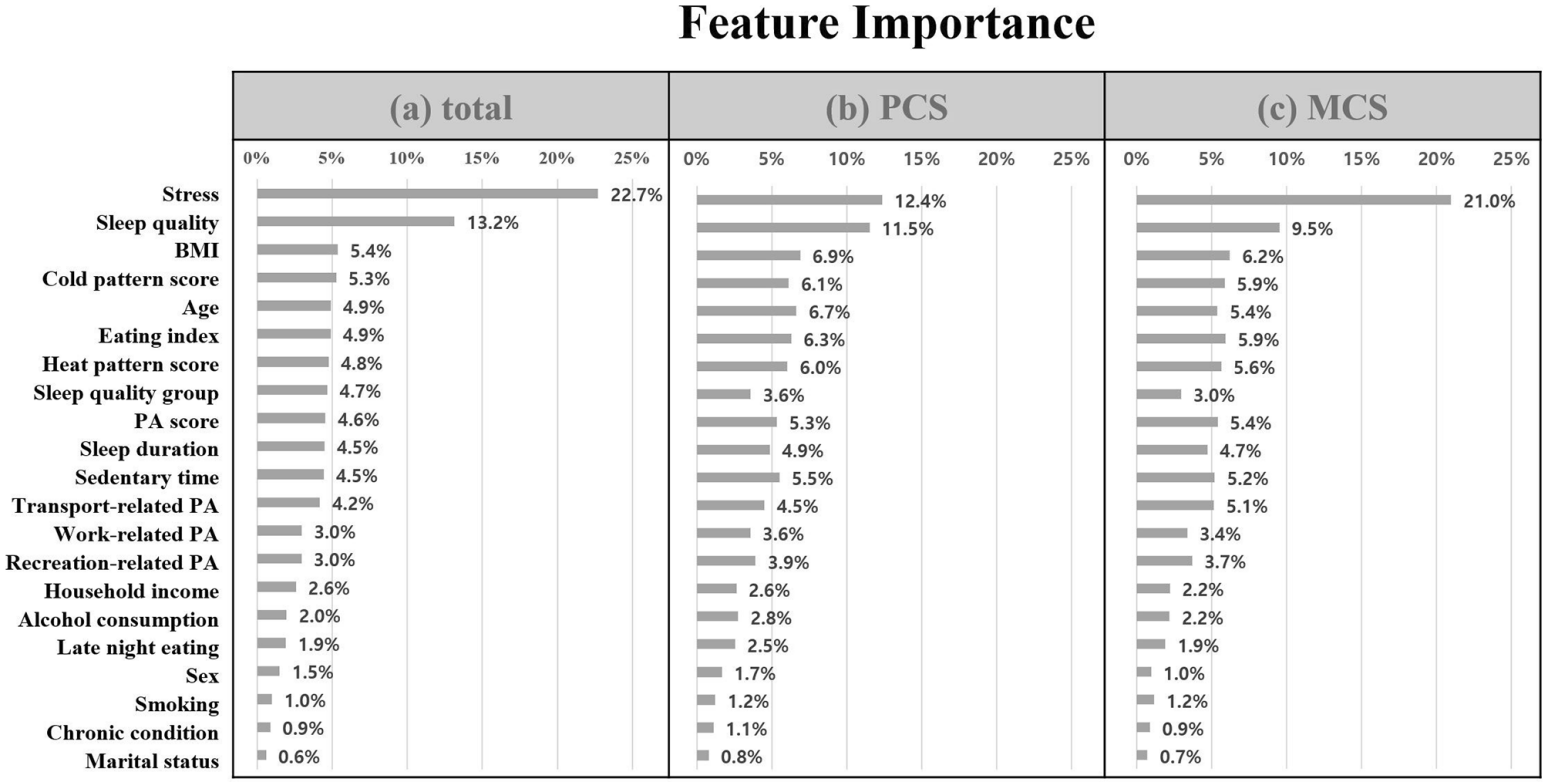
Feature importance for predicting QOL. Feature importance in a random forest model for total QOL (**a**), PCS (**b**), MCS (**c**) (x-axis). Order of features on the y-axis is listed in order of importance for total QOL. PCS, Physical component score; MCS, Mental component score; BMI, body mass index; PA, physical activity

Figure 2 illustrates the degree of importance of each variable for predicting QOL. For total QOL, stress and sleep quality were significant features of high importance, followed by BMI, cold pattern score, eating index, physical activity, and sleep. Stress and sleep quality were significant features of high importance for predicting PCS and MCS. The order of importance of each variable for predicting PCS and MCS was similar to that for total QOL, but the degree of influence differed.

### Visualization of feature importance

**Fig. 3:**
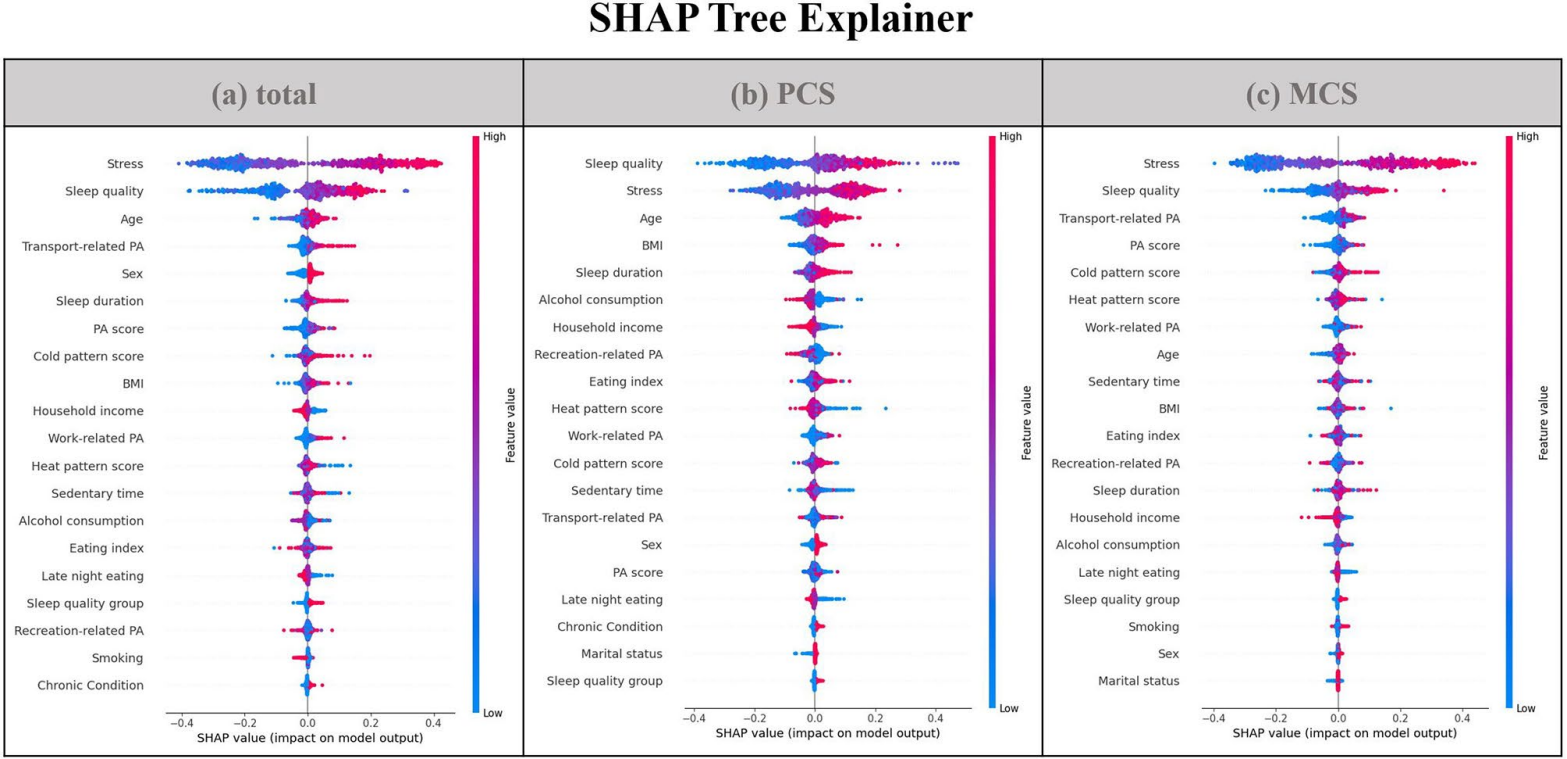
Visualization of feature importance using the SHAP. Summary plot where features appear in order of their sum of SHAP value magnitudes for total QOL (**a**), PCS (**b**), and MCS (**c**). Feature ranking (y-axis) is the order of importance of a feature in a prediction model. SHAP values (x-axis) indicate the predictive power of the prediction model. Each row is a plot of the influence on each validation data as dots. Red dots (high) represent the degree of prediction of “low QOL,” and blue dots (low) represent the degree of prediction of “high QOL.” PCS, Physical component score; MCS, Mental component score; PA, physical activity; BMI, body mass index

Figure 3 visualizes the influence of 21 variables on predicting the three types of QOL, using SHAP. Each row plots the influence of each feature on the validation data as dots. The greater the absolute SHAP value, the more important the feature is in predicting QOL. Stress was the most potent predictor of total QOL and MCS, followed by sleep quality and physical activity. The most potent predictor of PCS was sleep quality, followed by stress and age. Further, QOL decreased with increasing overall stress and poor sleep quality, and this was expressed as red dots (high), indicating the degree of prediction of low QOL.

## Discussion

This study generated QOL prediction models for use among middle-aged adults in South Korea using ML and analyzed the differences in the influence of each variable. We predicted QOL deterioration using individual factors linked to QOL and modifiable multifaceted lifestyle practices. A random forest model showed good performance. Although the feature importance varied depending on the target index, stress and sleep quality were major features. Further, applying SMOTE enhanced the performance of QOL prediction models, particularly the random forest. Our study utilizes explainable artificial-intelligence (XAI) techniques to provide detailed evidence on the effects of positive lifestyle changes on the quality of life of middle-aged adults—an age group at an important stage in life in terms of aging well. We expect that our findings will contribute to developing a health management model that induces changes by prioritizing the influence of various daily lifestyle practices to promote better QOL, such as promoting self-management and establishing systems to detect health change.

In our study, the random forest model showed good performance in predicting three QOL health indices. Models using 21 variables without applying SMOTE showed good performance with an F1-score ranging from 0.724 to 0.792 for total QOL, 0.653–0.719 for PCS, and 0.674–0.750 for MCS. These results were similar to the performance reported in previous studies. A study that predicted the QOL of older adults using ML also reported a model with an accuracy of 0.93 and an F-score of 0.49 [13]. The differences in performance indicators across studies are presumed to be attributable to differences in participant data and QOL indices chosen as targets for prediction with ML models.

In terms of feature importance, stress and sleep quality were identified as significant features for predicting QOL. Although feature importance varied depending on the target index (PCS, MCS, total QOL), two features were identified as important. A study that analyzed older adults with chronic diseases using nationally representative survey data showed that monthly income, diagnosis of chronic disease, depression, discomfort, and perceived health status are five significant factors associated with health-related QOL among older adults [13]. Another study reported that confidence, level of self-care, and acceptance of chronic disease are some factors that predict health status and, ultimately, QOL [33]. Further, a study on low perceived QOL among medical students reported that poor sleep quality is associated with poor QOL in a multiple linear regression analysis using questionnaire data [34]. Although these studies differ from ours, which used data collected from middle-aged adults, the findings show the importance of sleep quality.

ML algorithms are useful in diagnosing various health conditions, and active research seeks to enhance their diagnostic performance [35]. We performed data synthesis via SMOTE to resolve data imbalance and confirmed that SMOTE could improve model performance. A study that predicted diabetes mellitus using healthcare data applied SMOTE to resolve data imbalances. The performance improved from 0.027 to 0.667 for the probabilistic neural network and 0.215 to 0.726 for the decision tree [36]. A study that used demographic, lifestyle, and blood test parameters to predict metabolic syndrome reported that model performance was enhanced up to an AUC of 0.091 after using SMOTE [37]. In our study, the F1-score for the random forest algorithm rose from 0.792 to 0.827 for total QOL, 0.719 to 0.777 for PCS, and 0.752 to 0.802 for MCS. Considering that clinical data frequently exhibit data imbalance across classes, data oversampling techniques, such as SMOTE, will be useful for developing diagnostic techniques, such as predicting QOL.

QOL is also associated with multiple factors. Our results showed that low QOL is associated with female sex, old age, high BMI, low income, presence of a chronic condition, non-smoking status, high cold–heat pattern score, high night snacking frequency, low eating index, low sleep quality, high physical activity, low sedentary lifestyle, moderate-/high-intensity work, high transport time, low leisure time, and high stress. These results are somewhat consistent with previous findings [5, 7]. Although the percentage of non-smokers was high in the low QOL group, this seems to pertain to the high percentage of women (74.4%) among non-smokers. Our study also confirmed the effects of cold–heat pattern identification—an individual characteristic—on QOL, and observed that it is an important factor in health [38].

Our study presents some points for improvement and limitations. A previous survey of QOL reported that physical functioning indices, such as grip strength, are correlated with QOL [39]. Therefore, adding physical functioning parameters to the basic demographic factors and lifestyle factors could enhance model performance. In addition, several studies are underway to interpret the results of ML model predictions, and such research is particularly valuable for clinical decision support systems [40]. As with our study, much research is being conducted on explainable AI; however, more research is needed to enhance the reliability of interpretation [41]. Finally, we analyzed the major predictors of QOL using cross-sectional data, but our analysis cannot infer the order between various factors and QOL. Despite these limitations, the representativeness of our study data was ensured by sex-, age-, and region-based population stratification, whereas the accuracy of assessment of various lifestyle factors was improved using standardized instruments with established validity and reliability.

## Conclusions

This study compared the performance of several QOL prediction models, using individual and lifestyle factors among middle-aged adults in South Korea. A tree-based ML algorithm, random forest, was identified to have high accuracy in predicting the three QOL indices of total QOL, PCS, and MCS. The performance of the models improved when SMOTE was applied. As a result of analysis using XAI, stress and sleep quality were identified as two major predictors of QOL among middle-aged adults. Interest in QOL is increasing amid population aging and advances in healthcare technology. It is important to develop health management programs that enhance QOL for middle-aged adults from a multidisciplinary perspective, and the use of artificial intelligence technologies such as XAI will be useful.

## Data Availability

The datasets are not available owing to confidentiality and ethical concerns. Further inquiries can be directed to the corresponding author or Korea Medicine Data Center (www.kdc.kiom.re.kr).
